# Effectiveness of Coenzyme Q10 Supplementation for Reducing Fatigue: A Systematic Review and Meta-Analysis of Randomized Controlled Trials

**DOI:** 10.3389/fphar.2022.883251

**Published:** 2022-08-24

**Authors:** I-Chen Tsai, Chih-Wei Hsu, Chun-Hung Chang, Ping-Tao Tseng, Ke-Vin Chang

**Affiliations:** ^1^ Institute of Clinical Medicine, National Yang Ming Chiao Tung University, Taipei, Taiwan; ^2^ Congenital Heart Disease Study Group, Asian Society of Cardiovascular Imaging, Seoul, Korea; ^3^ InnovaRad Inc., Taichung, Taiwan; ^4^ Department of Psychiatry, Kaohsiung Chang Gung Memorial Hospital and Chang Gung University College of Medicine, Kaohsiung, Taiwan; ^5^ Department of Computer Science and Information Engineering, National Cheng Kung University, Tainan, Taiwan; ^6^ Institute of Clinical Medical Science, China Medical University, Taichung, Taiwan; ^7^ Department of Psychiatry and Brain Disease Research Center, China Medical University Hospital, Taichung, Taiwan; ^8^ An Nan Hospital, China Medical University, Tainan, Taiwan; ^9^ Prospect Clinic for Otorhinolaryngology and Neurology, Kaohsiung, Taiwan; ^10^ Institute of Biomedical Sciences, National Sun Yat-sen University, Kaohsiung, Taiwan; ^11^ Department of Psychology, College of Medical and Health Science, Asia University, Taichung, Taiwan; ^12^ Department of Physical Medicine and Rehabilitation, National Taiwan University Hospital and National Taiwan University College of Medicine, Taipei, Taiwan; ^13^ Department of Physical Medicine and Rehabilitation, National Taiwan University Hospital, Bei-Hu Branch, Taipei, Taiwan; ^14^ Center for Regional Anesthesia and Pain Medicine, Wang-Fang Hospital, Taipei Medical University, Taipei, Taiwan

**Keywords:** coenzyme Q10, fatigue, clinical trials, meta-analysis, systematic review

## Abstract

Coenzyme Q10 (CoQ10) is a popular nutritional supplement, an antioxidant and an essential component of the mitochondrial electron transport chain. Several clinical studies have suggested that fatigue can be reduced by antioxidant supplementation. However, the data on this topic has been sparse to date. Hence, we conducted this meta-analysis with the aim of investigating the effectiveness of fatigue reduction via CoQ10 supplementation. More specifically, we searched electronic databases for randomized controlled trials (RCTs) published from the database inception to January 2022. A random effects model was implemented to conduct the meta-analysis among 13 RCTs (with a total of 1,126 participants). As compared with the placebo groups evaluated in each RCT, the CoQ10 group showed a statistically significant reduction in fatigue scores (Hedges’ *g* = −0.398, 95% confidence interval = −0.641 to −0.155, *p* = 0.001). The directions of the treatment effects were consistent between the healthy and diseased participants. Compared with the placebo group, the effect of reducing fatigue was statistically significant in the subgroup using the CoQ10-only formulation but not in the subgroup using CoQ10 compounds. The results of our meta-regression demonstrate that increases in the daily dose (coefficient = −0.0017 per mg, *p* < 0.001) and treatment duration (coefficient = −0.0042 per day, *p* = 0.007) of CoQ10 supplementation were correlated with greater fatigue reduction. There was only one adverse (gastrointestinal) event in the 602 participants who underwent the CoQ10 intervention. Based on the results of this meta-analysis, we conclude that CoQ10 is an effective and safe supplement for reducing fatigue symptoms.

**Systematic Review Registration:** https://inplasy.com/inplasy-2022-1-0113/, identifier INPLASY202210113

## 1 Introduction

Fatigue is a symptom that occurs in both healthy and diseased individuals (Finsterer and Mahjoub, 2014). This symptom is described as unusual overwhelming tiredness that cannot be explained by physiological exhaustion in the wake of physical or mental efforts and that is not sufficiently recovered by regular rest and sleep (Haß et al., 2019). In the general population, the prevalence of temporary fatigue ranges from 4 to 45%, while that of chronic fatigue (i.e., fatigue lasting for >6 months) ranges from 2 to 11% (Cathébras et al., 1992; Ridsdale et al., 1993; Jason et al., 1999; Cullen et al., 2002). Fatigue is also common in patients with poliomyelitis (Peel et al., 2015) and multiple sclerosis (Sanoobar et al., 2016; Moccia et al., 2019) as well as in cancer patients undergoing chemotherapy (Iwase et al., 2016). The annual total cost of productivity loss due to chronic fatigue syndrome in the United States alone is approximately US$ 9.1 billion, which is roughly equal to US$ 20,000 per resident (Reynolds et al., 2004). Although the etiology of fatigue remains poorly understood (Filler et al., 2014), mitochondrial dysfunction (Filler et al., 2014) and pro-inflammatory status (Haß et al., 2019) may play a role. Fortunately, fatigue can be rigorously measured and is potentially treatable (Finsterer and Mahjoub, 2014).

Coenzyme Q10 (CoQ10) is a popular nutritional supplement and a lipid-soluble antioxidant that is endogenously produced by the human body. It is also an essential component of the mitochondrial electron transport chain (Aaseth et al., 2021). Case-control studies conducted by Maes et al. showed that, as compared with healthy subjects, patients with chronic fatigue syndrome have lower plasma levels of CoQ10 (Maes et al., 2009a; Maes et al., 2009b). A statistically significant inverse relationship has also been found between CoQ10 levels and fatigue severity (Maes et al., 2009a). Thus, CoQ10 supplementation has been successfully applied for reducing fatigue in patients with various conditions, including chronic fatigue syndrome (Castro-Marrero et al., 2015; Castro-Marrero et al., 2016; Fukuda et al., 2016; Castro-Marrero et al., 2021) and fibromyalgia (Cordero et al., 2013; Miyamae et al., 2013; Di Pierro et al., 2017), as well as in healthy subjects (Morikawa et al., 2019; Mizuno et al., 2020). However, inconsistencies in clinical outcomes have been identified across different trials. Therefore, in the current study, we performed a systematic review and meta-analysis to investigate the effects of CoQ10 treatment on fatigue symptoms and syndromes.

## 2 Materials and Methods

### 2.1 General Guidelines

We followed the guidelines delineated in the latest version of the PRISMA 2020 guidelines (Supplementary Table S1) to conduct this meta-analysis (Page et al., 2021a). This study, which was registered in INPLASY with the registration number INPLASY202210113 (Tsai and Chang, 2022), did not require ethics review board approval or participant informed consent.

### 2.2 Database Searches and the Identification of Eligible Manuscripts

Two authors (I-CT and K-VC) conducted independent electronic searches in the PubMed, Embase, Cochrane CENTRAL, Web of Science, and ClinicalTrials.gov databases using the following keywords (“Q10” OR “Q 10” OR “CoQ10” OR “Coenzyme Q10” OR “ubiquinol-10” OR “ubiquinol” OR “ubiquinone”) AND (“fatigue” OR “chronic fatigue syndrome” OR “tiredness”). The search was conducted from the inception of each database (i.e., the earliest record) to the date of the database search (16 January 2022). We note that ubiquinone is the fully oxidized form of CoQ10, and ubiquinol is the reduced form. These two forms are continually interconverted in the body and have similar bioactivities (Mantle and Dybring, 2020). The detailed search strategy for this systematic review and meta-analysis is provided in the Supplementary Material (Supplementary Table S2).

Initially, the two authors responsible for conducting this search screened the identified titles and abstracts for eligibility through a consensus process. The PubMed and EMBASE databases were thoroughly scrutinized for any potentially eligible trials. We also checked the reference lists of an identified review article (Mehrabani et al., 2019) and performed additional manual searches. A third reviewer and study author (P-TT) was consulted for situations in which the two aforementioned authors could not reach a consensus. No language restrictions were applied to this search.

### 2.3 Inclusion and Exclusion Criteria

The PICO (population, intervention, comparison, outcome) setting of the current meta-analysis was as follows: P: human participants; I: CoQ10 supplementation; C: placebo; and O: changes in fatigue symptom scores.

The following inclusion criteria were used: 1) randomized controlled trials (RCTs) enrolling human participants, 2) RCTs investigating the quantitative evaluation of fatigue symptoms before and after CoQ10 supplementation, 3) placebo-controlled trials (without age or treatment duration limitations), and 4) trials with available data for pre- and post-intervention fatigue assessments or evaluations of changes in fatigue scores. Open-label studies were also included in this meta-analysis as recent studies have found that open-label placebos had similar efficacy to double-blind placebos (Lembo et al., 2021; von Wernsdorff et al., 2021).

The exclusion criteria for this review and meta-analysis were as follows: 1) non-RCTs, 2) studies focusing on athletic muscle exhaustion rather than generalized fatigue, 3) studies lacking a placebo-controlled group, 4) studies lacking quantitative assessments of fatigue, and 5) studies enrolling participants that overlapped with a previously published trial.

### 2.4 Methodological Quality Appraisal

To investigate the methodological quality of the evaluated studies, we used the Cochrane risk of bias tool for randomized trials (version 2, RoB 2, London, United Kingdom), which consists of six main items for evaluating study quality: the randomization process, intervention adherence, missing outcome data, outcome measurement, selective reporting, and the overall risk of bias (Sterne et al., 2019).

In the intervention adherence section of the RoB 2, there are two options presented for literature assessment: intention-to-treat (intervention assignment) and per-protocol (intervention adherence). In this meta-analysis, we chose the per-protocol evaluation (Sterne et al., 2019) since it fits best with the design of our included studies.

### 2.5 Primary Outcome (Fatigue Score Change)

The primary outcomes evaluated in this investigation were changes in fatigue scores following CoQ10 supplementation or placebo regimens. We also examined the validity and appropriateness of the fatigue scale used in each trial (Fisk et al., 1994; Chandran et al., 2007; Fukuda et al., 2008). If there was more than one scoring system for fatigue evaluation implemented in a single trial, the index test included in the meta-analysis was decided by author consensus (I-CT and K-VC).

### 2.6 Secondary Outcome (Treatment-Associated Adverse Event Rates)

The secondary outcome evaluated in this investigation was the treatment-associated adverse event rate. For cells with zero events, zero was replaced by 0.5 to enable calculation (Deeks et al., 2021). The aforementioned outcomes were quantified using odds ratios.

### 2.7 Data Extraction and Management

Two independent authors (I-CT and K-VC) extracted data from the evaluated studies, including demographic data, study design parameters, details of the administered CoQ10 and placebo regimens, and the primary and secondary outcome values. To avoid misinterpretation, the evaluators paid special attention to the effect direction of the scale used in each trial. In situations where data were unavailable within the published articles, we contacted the corresponding authors to obtain the original data.

Data extraction and conversion as well as merging the results from various study arms using different CoQ10 dosages were processed according to the recommendations of the Cochrane Handbook for Systematic Reviews of Interventions and the associated medical literature (Hozo et al., 2005; Higgins et al., 2021a; Higgins et al., 2021b). If post-treatment data were available at multiple time points, we extracted the outcome reported at the end of the intervention for statistical analysis. For crossover studies, we included only the first study interval to avoid carry-over effects (Higgins et al., 2021a).

### 2.8 Statistical Analyses

Because of the heterogeneity of the target populations within the enrolled studies, the current meta-analysis was conducted with a random-effects model (Borenstein et al., 2009) implemented using Comprehensive Meta-Analysis software (version 3, Biostat, Englewood, NJ, United States). A two-tailed *p* value of less than 0.05 was considered statistically significant.

We used Hedges’ *g* and 95% confidence intervals (CIs) to quantify the primary study outcomes (i.e., changes in fatigue scores). Hedges’ *g* values of 0.2, 0.5, and 0.8 were considered small, moderate, and large effect sizes, respectively (Hedges, 1981). Odds ratios (ORs) and their associated 95% CIs were evaluated to investigate secondary outcomes (i.e., treatment-associated adverse event rates).

*I* ^*2*^ and Cochran’s *Q* statistics were also examined to evaluate the degree of heterogeneity across studies. *I* ^*2*^ values of 25, 50, and 75% were considered low, moderate, and high heterogeneity, respectively (Higgins et al., 2003). We likewise performed subgroup analyses based on disease and CoQ10 formulations. Meta-regression analyses regarding the treatment effects of daily CoQ10 doses as well as evaluations of various treatment durations were conducted to determine whether the fatigue-alleviating effects of CoQ10 correlated with the aforementioned parameters.

To confirm the robustness of this meta-analysis, sensitivity analyses were performed using the one-study removal method to determine whether there was a statistically significant change in the summary effect size after removing a particular trial from the analysis (Deeks et al., 2021).

Potential publication bias was evaluated using guidelines set forth by the Cochrane Handbook for Systematic Reviews of Interventions (Page et al., 2021b). Funnel plots were generated and visually inspected. Egger’s regression tests were implemented when 10 or more datasets were available.

## 3 Results

### 3.1 Study Identification and Selection

**FIGURE 1:**
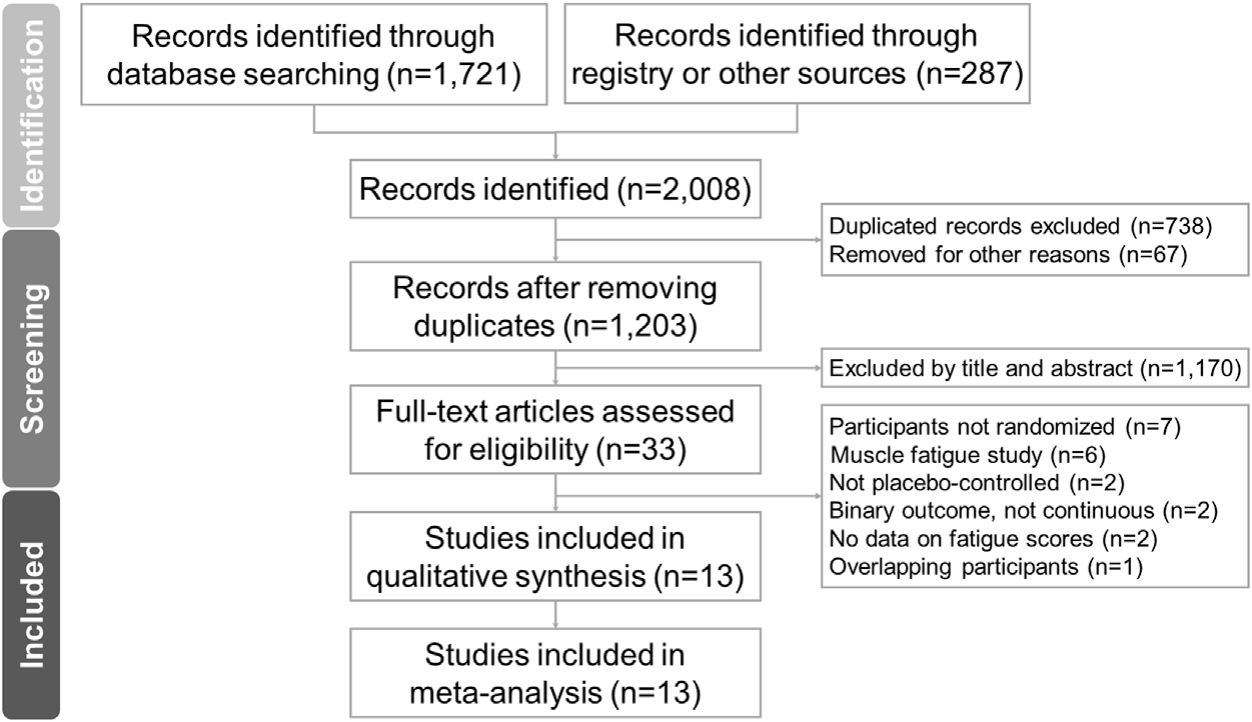
PRISMA 2020 flowchart for the current meta-analysis.

The PRISMA flowchart for the literature search is shown in Figure 1. After removing duplicate articles and excluding non-relevant articles by an examination of titles and abstracts, we ultimately included 13 RCTs in the final analysis (Berman et al., 2004; Lee et al., 2011; Cordero et al., 2013; Lesser et al., 2013; Castro-Marrero et al., 2015; Fukuda et al., 2015; Peel et al., 2015; Sanoobar et al., 2016; Di Pierro et al., 2017; Morikawa et al., 2019; Mizuno et al., 2020; Mousavi et al., 2020; Castro-Marrero et al., 2021). The articles excluded in the final stage and the reasons for exclusion are listed in Supplementary Table S3 (Singh et al., 2003; Langsjoen et al., 2005; Kumar et al., 2007; Mizuno et al., 2008; Gökbel et al., 2010; Cordero et al., 2012a; Cordero et al., 2012b; Fedacko et al., 2013; Gharahdaghi et al., 2013; Miyamae et al., 2013; Castro-Marrero et al., 2016; Fukuda et al., 2016; Iwase et al., 2016; Menon et al., 2017; Langsjoen et al., 2019; Moccia et al., 2019; Negro et al., 2019; Gomez-Centeno et al., 2020; Schweiger et al., 2020; Suzuki et al., 2021). Details of data extraction from included randomized controlled trials are summarized in Supplementary Table S4.

The 13 eligible RCTs encompassed a total of 1,126 participants with a mean age of 49.3 ± 12.6 (standard deviation) years, of whom 25.6% (*n* = 288) were male. The study duration ranged from four (Mousavi et al., 2020) to 24 weeks (Lesser et al., 2013). Subject diagnoses included chronic fatigue syndrome (Castro-Marrero et al., 2015; Castro-Marrero et al., 2021), fibromyalgia (Cordero et al., 2013; Di Pierro et al., 2017), end-stage heart failure (Berman et al., 2004), obesity (Lee et al., 2011), breast cancer (with patients undergoing chemotherapy) (Lesser et al., 2013), end-stage renal disease (Fukuda et al., 2015), poliomyelitis (Peel et al., 2015), and multiple sclerosis (Sanoobar et al., 2016), and we also evaluated healthy subjects (Morikawa et al., 2019) and healthy individuals with fatigue (Mizuno et al., 2020; Mousavi et al., 2020). The details of the retrieved trials are summarized in Table 1.

**TABLE 1:** Summary of the retrieved trials investigating the effect of CoQ10 on reducing fatigue in the enrolled participants.

First author (year)	Country	Population	Participants (F/M)	Age^a^	Study design	Allocation concealment	Randomization	Funding/grants/support
Berman (2004)	Israel	End-stage heart failure	Total: 4/28^b^	54.6 (40-67)^b^	RCT, double-blind	External party	Not mentioned	N/A
Lee (2011)	Korea	Obesity	CoQ10: 15/11^b^ Placebo: 15/10	42.7±11.3^b^ 42.5±11.2	RCT, double-blind	Not mentioned	Not mentioned	N/A
Cordero (2013)	Spain	Fibromyalgia	CoQ10: 10/0^b^ ^,^ ^c^ Placebo: 10/0	44.3±9.7^b^ ^,^ ^c^ 55.0±5.0	RCT, double-blind	Not mentioned	Not mentioned	N/A
Lesser (2013)	USA	Breast cancer under chemotherapy	CoQ10: 122/0^b^ Placebo: 114/0	52 (31-85)^b^ 50 (28-72)	RCT, double-blind	Not mentioned	Stratified block	• National Cancer Institute
Castro-Marrero (2015)	Spain	Chronic fatigue syndrome	CoQ10+NADH: 39/0^b^ ^,^ ^c^ Placebo: 34/0	49.3±7.1^b^ ^,^ ^c^	RCT, double-blind	Not mentioned	Not mentioned	• Vitae Natural Nutrition, S.L.
Fukuda (2015)	Japan	End-stage renal disease	CoQ10 mix drink: 17/70^c^ Placebo: 15/71	55.6±10.0^c^ 56.2±8.9	RCT, double-blind	Coordination center	Stratified	• Asahi Kasei Kuraray Medical Cooperation• Ministry of Education, Culture, Sports, Science and Technology (Japan)• Health Labor Sciences Research Grant (Japan)
Peel (2015)	Australia	Poliomyelitis	CoQ10: 39/15^b^ ^,^ ^d^ Placebo: 32/17	69.9±8.4^b^ 69.8±8.2	RCT, double-blind	Coordination center	Matrix sequence	• Cancer and Polio Research Fund (UK)
Sanoobar (2016)	Iran	Multiple sclerosis	CoQ10: 20/2^c^ Placebo: 21/2	33.1±7.6^c^ 30.9±7.7	RCT, double-blind	Not mentioned	Not mentioned	• Tehran University of Medical Sciences
Di Pierro (2017)	Italy	Fibromyalgia	CoQ10: 12/0^b^ ^,^ ^c^ Placebo: 10/0	52.5±10.4^b^ ^,^ ^c^ 53.6±7.8	RCT, open-label	Coin tossing	Coin tossing	N/A
Morikawa (2019)	Japan	Healthy subjects	CoQ10: 16/14^b^ Placebo: 16/14	40.5±7.3^b^ 42.9±7.3	RCT, double-blind	Not mentioned	Stratified block	• Kaneka, Inc.
Mizuno (2020)	Japan	Healthy subjects with fatigue	CoQ10: 28/14^b^ ^,^ ^c^ Placebo: 13/7	42.1±10.9^b^ ^,^ ^c^ 41.3±13.4	RCT, double-blind	Not mentioned	Stratified	• Kaneka, Inc.• Japan Science and Technology Agency
Mousavi (2020)	Iran	Health subjects (nurses) with fatigue	CoQ10: 47/7^c^ Placebo: 45/6	35.1±8.1^c^ 35.7±8.9	RCT, double-blind	Assignment double-blinded	Permuted block	• Isfahan University of Medical Sciences
Castro-Marrero (2021)	Spain	Chronic fatigue syndrome	CoQ10+NADH: 72/0^c^ Placebo: 72/0	45.38±7.81^c^ 46.79±6.48	RCT, double-blind	Independent investigator	Random number	• Vitae Health Innovation• Vall d’Hebron Hospital Research Institute

CoQ10, coenzyme Q10; NADH, reduced form of nicotinamide adenine dinucleotide; RCT, randomized controlled trial; UK, United Kingdom; USA, United States of America.

a Age is presented as means ± standard deviations or as medians (ranges).

b Allocated participants.

c Per-protocol participants.

d The total sample size of the CoQ10 group was retrieved from data presented in Figure 1 and Table 2 due to inconsistency in Table 1.

Three studies evaluated compounds mixed with CoQ10. More specifically, CoQ10 with a reduced form of nicotinamide adenine dinucleotide (NADH) was evaluated in two of these trials (Castro-Marrero et al., 2015; Castro-Marrero et al., 2021) and CoQ10 in a multi-vitamin nutritional drink was evaluated in the other trial (Fukuda et al., 2015). Ten studies evaluated CoQ10 only (Berman et al., 2004; Lee et al., 2011; Cordero et al., 2013; Lesser et al., 2013; Peel et al., 2015; Sanoobar et al., 2016; Di Pierro et al., 2017; Morikawa et al., 2019; Mizuno et al., 2020; Mousavi et al., 2020). The intervention details, tools for fatigue assessment, adverse events, and study withdrawals are summarized in Table 2.

**TABLE 2:** Summary of the CoQ10 interventions administered in the study treatment arms of the retrieved trials.

First author(year)	Population	Duration	CoQ10 product/manufacturer	Daily CoQ10 dose (per-protocol N)	Control (per-protocol N)	Fatigue outcome measurement (score range)	AE associated with CoQ10 withdrawal
Berman (2004)	End-stage heart failure	3 months	Ultrasome capsules/Herbamed Ltd. (Israel)	60 mg/day (13)	Matching placebo (14)	Minnesota Living with Heart Failure Questionnaire fatigue score (0-5)	One ultrasome-induced intestinal upset withdrawal
Lee (2011)	Obesity	12 weeks	Ubiquinone/Daewoong Pharmacy (Korea)	200 mg/day (17)	Matching placebo (19)	Fatigue Severity Scale (9-63)	No
Cordero (2013)	Fibromyalgia	40 days	CoQ10 capsules/Pharma Nord (Denmark)	300 mg/day (10)	Matching placebo (10)	Fibromyalgia Impact Questionnaire fatigue score (0-10)	No
Lesser (2013)	Breast cancer under chemotherapy	24 weeks	CoQ10/Soft Gel Technologies (USA)	300 mg/day (78)	Matching placebo (61)	Profile of Mood States fatigue subscale (0-4)	No. All AE-related withdrawals were due to chemotherapy based on the investigators’ review.
Castro-Marrero (2015)	Chronic fatigue syndrome	8 weeks	ReConnect/Vitae Natural Nutrition (Spain)	200 mg/day with 20 mg/day NADH (39)	Matching placebo (34)	Fatigue Impact Scale (0-160)	No
Fukuda (2015)	End-stage renal disease	12 weeks	AMP01/Asahi Kasei Kuraray Medical Corporation (Japan)	Nutritional drink with 30 mg/day CoQ10 (87)	Matching placebo (86)	Fatigue Scale (0-32)	No. All AE and withdrawals were not associated with the study intervention (which was conducted by a safety monitoring board). AE-associated withdrawal: CoQ10 6/97, placebo 5/99
Peel (2015)	Poliomyelitis	60 days	CoQ10 capsules/Health World Limited (Australia)	100 mg/day (54)^a^	Matching placebo (49)	Multidimensional Assessment of Fatigue (1-50)	No. All AEs were not associated with CoQ10 supplementation. AE: CoQ10 7/54, placebo 5/49. AE-associated withdrawal: CoQ10 5/54, placebo 2/49
Sanoobar (2016)	Multiple sclerosis	12 weeks	CoQ10 capsules/not mentioned	500 mg/day (22)	Matching placebo (23)	Fatigue Severity Scale (9-63)	No
Di Pierro (2017)	Fibromyalgia	3 months	DDM Chinone® sachets/Labomar (Italy)	400 mg/day (12)	Comparable placebo (10)	Functional Assessment of Chronic Illness Therapy (0-44)	No
Morikawa (2019)	Healthy subjects	8 weeks	Uniquinol soft capsules/Kaneka, Inc. (Japan)	100 mg/day (24)	Matching placebo (28)	Fatigue Visual Analogue Scale (0-10)	No. All AEs were not associated with the treatment. AE: CoQ10 8/30, placebo 6/30
Mizuno (2020)	Healthy individuals with fatigue	12 weeks	Uniquinol soft capsules/Kaneka, Inc. (Japan)	150 mg/day (22) 100 mg/day (20)	Matching placebo (20)	Fatigue Visual Analogue Scale (0-100)	No
Mousavi (2020)	Nurses with fatigue	4 weeks	CoQ10 capsules/Nutri Century (Canada)	200 mg/day (54)	Matching placebo (51)	Nurses' fatigue scale (21-105)	No
Castro-Marrero (2021)	Chronic fatigue syndrome	8 weeks	ReConnect/Vitae Health Innovation (Spain)	200 mg/day with 20 mg/day NADH (72)	Matching placebo (72)	Fatigue Impact Scale (0-160)	No. All AEs were not associated with the treatment. AE-associated withdrawal: CoQ10+NADH 8/104, placebo 11/103

AE, adverse events; CoQ10, coenzyme Q10; NADH, reduced form of nicotinamide adenine dinucleotide; USA, United States of America

a The total number of subjects enrolled in the CoQ10 group was retrieved based on data reported in Figure 1 and Table 2, due to inconsistency in Table 1.

### 3.2 Methodological Quality of the Included Studies

**FIGURE 2:**
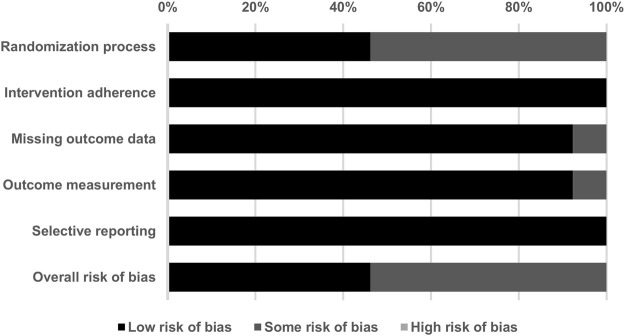
Summary of quality assessment for the studies included in the current meta-analysis using version 2 of the Cochrane risk-of-bias tool for randomized trials.

With respect to the overall methodological quality of the included studies, we found that 46.2% of the evaluated studies had a low risk of bias, 53.8% had some risk of bias, and 0% had a high risk of bias (Figure 2). In a detailed assessment, seven studies (Lee et al., 2011; Cordero et al., 2013; Lesser et al., 2013; Castro-Marrero et al., 2015; Sanoobar et al., 2016; Morikawa et al., 2019; Mizuno et al., 2020) were rated as having “some” risk of bias in the randomization process because they did not reveal details of the allocation concealment. One study (Lee et al., 2011) was rated as having “some” risk of bias with regard to missing outcome data, since the study excluded five subjects from the CoQ10 group due to an unchanged level of serum CoQ10 after supplementation. One study (Di Pierro et al., 2017) was rated as having “some” risk of bias in the outcome measurement because it was an open-label study and the participants were aware of the interventions they received. The details of the risk of bias assessment are summarized in Table 3.

**TABLE 3:** Detailed quality assessment of the included studies using the Cochrane risk-of-bias tool, version 2.

First author (year)	Randomization process	Intervention adherence	Missing outcome data	Outcome measurement	Selective reporting	Overall RoB
Berman (2004)	L	L	L	L	L	L
Lee (2011)	S^a^	L	S^b^	L	L	S
Cordero (2013)	S^a^	L	L	L	L	S
Lesser (2013)	S^a^	L	L	L	L	S
Castro-Marrero (2015)	S^a^	L	L	L	L	S
Fukuda (2015)	L	L	L	L	L	L
Peel (2015)	L	L	L	L	L	L
Sanoobar (2016)	S^a^	L	L	L	L	S
Di Pierro (2017)	L	L	L	S^c^	L	L
Morikawa (2019)	S^a^	L	L	L	L	S
Mizuno (2020)	S^a^	L	L	L	L	S
Mousavi (2020)	L	L	L	L	L	L
Castro-Marrero (2021)	L	L	L	L	L	L

CoQ10, coenzyme Q10; H, high risk of bias; L, low risk of bias; RoB, risk of bias; S, some risk of bias

a These studies didn't provide allocation concealment details.

b This study excluded five subjects from the CoQ10 group because their blood levels of CoQ10 failed to increase over the study course.

c An open-label study. The participants were aware of the intervention they received.

### 3.3 Primary Outcome: Effects of CoQ10 Supplementation on Fatigue

**FIGURE 3:**
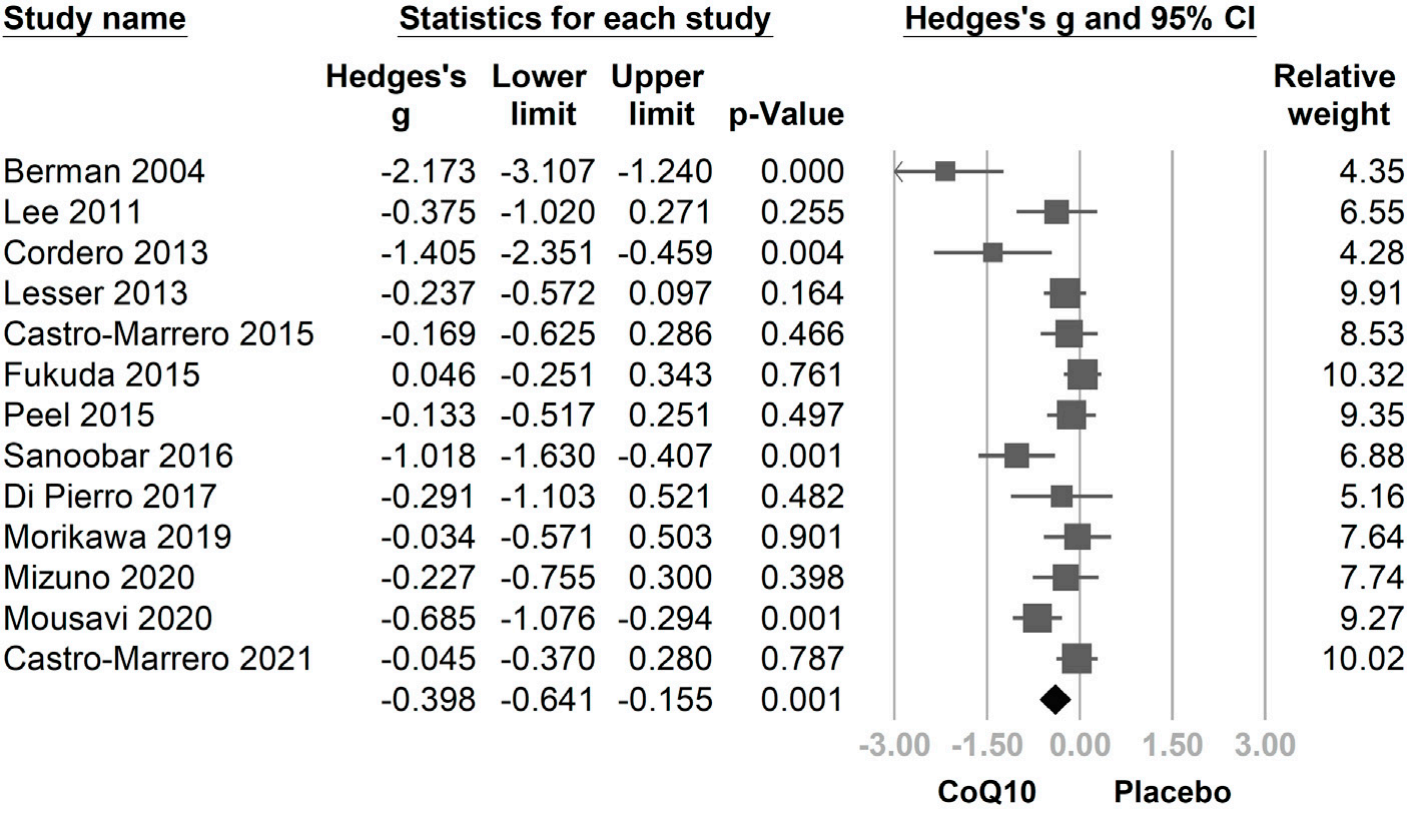
Forest plot of the effects of oral coenzyme Q10 (CoQ10) on fatigue as compared with the placebo. CoQ10 was found to be effective in reducing fatigue. CI, confidence interval.

**FIGURE 4:**
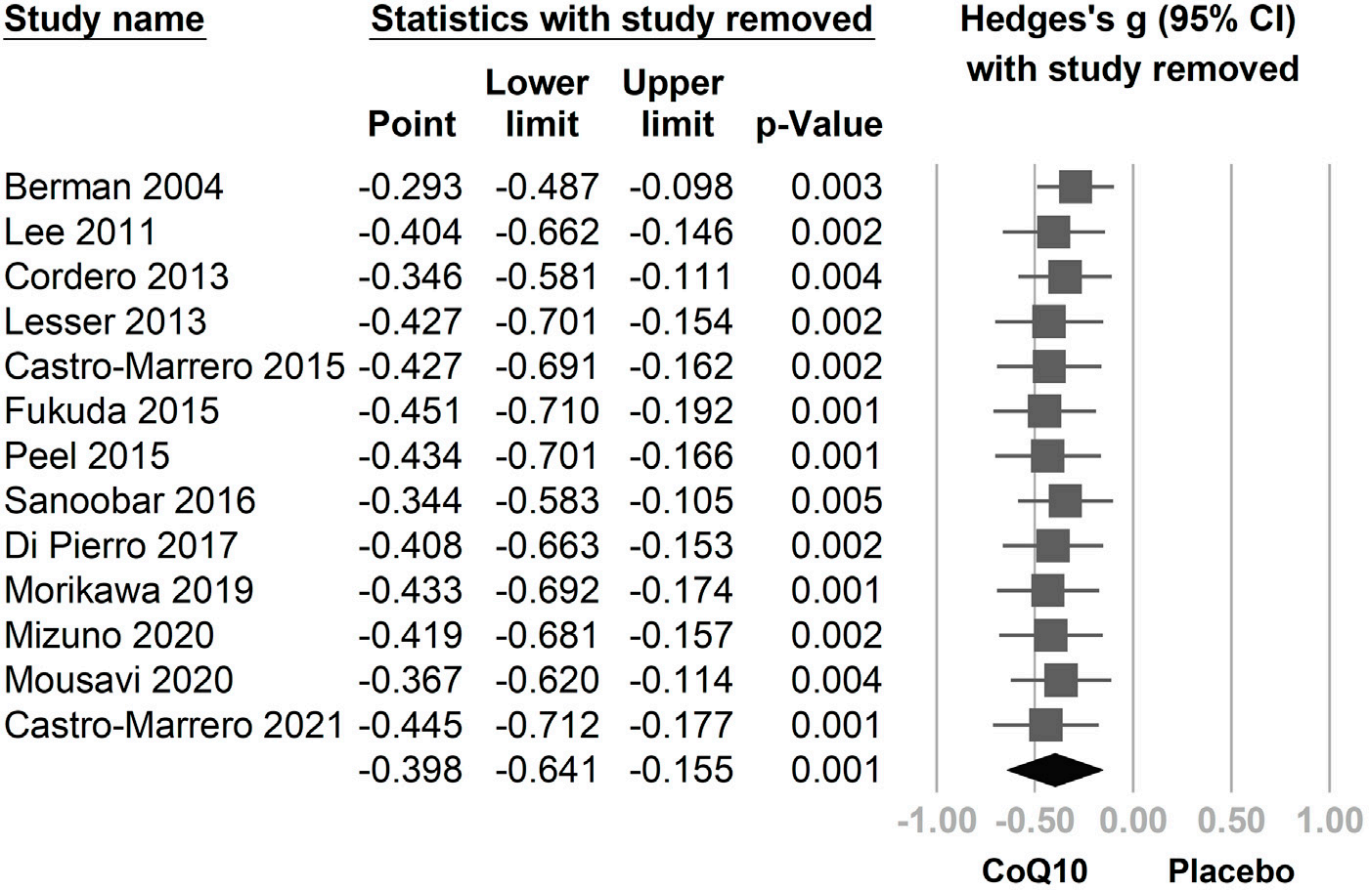
The results of a sensitivity analysis using the one-study removal method. The main result did not change significantly after removing any one of the included trials. All analyses showed statistically significant effects of CoQ10 in relieving fatigue. CI, confidence interval.

In the combined 13 trials (Figure 3), CoQ10 demonstrated a statistically significant reduction with regard to fatigue symptomology (Hedges’ *g* = −0.398, 95% CI = −0.641 to −0.155, *p* = 0.001, *I* ^*2*^ = 69.5%). However, moderate-to-high heterogeneity was observed. A sensitivity analysis was performed using the one-study removal method. The results showed a consistently statistically significant effect of CoQ10 on fatigue reduction. The summary effect sizes did not change the statistical significance of these findings when any of the included studies were removed (Figure 4).

**FIGURE 5:**
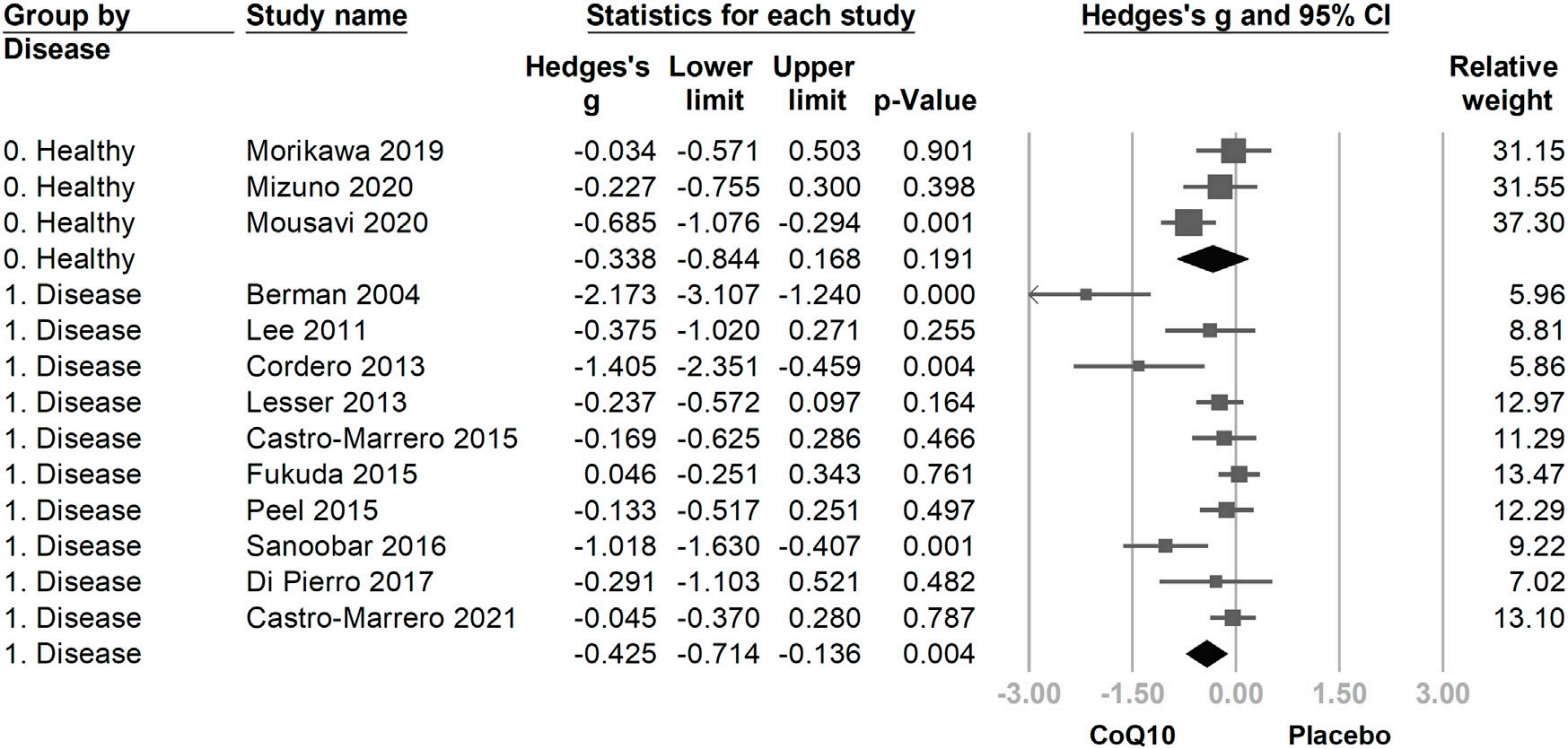
The forest plot of subgroup analysis using the participants’ condition as the moderator, including healthy participants and patients with disease. The directions of association between the use of coenzyme Q10 (CoQ10) and fatigue assessment were consistent in the subgroups, with overlapping 95% confidence intervals (CIs).

We then divided the included trials into two subgroups: healthy participants (Morikawa et al., 2019; Mizuno et al., 2020; Mousavi et al., 2020) and patients with a fatigue-associated disease (fibromyalgia (Cordero et al., 2013; Di Pierro et al., 2017), chronic fatigue syndrome (Castro-Marrero et al., 2015; Castro-Marrero et al., 2021), heart failure (Berman et al., 2004), obesity (Lee et al., 2011), breast cancer (Lesser et al., 2013), end-stage renal disease (Fukuda et al., 2015), poliomyelitis (Peel et al., 2015), multiple sclerosis (Sanoobar et al., 2016)). The direction of association between the use of CoQ10 and fatigue assessment was consistent in the subgroups of healthy participants (Hedges’ *g* = −0.351, 95% CI = −0.756 to 0.053, *p* = 0.089) and in patients with disease (Hedges’ *g* = −0.433, 95% CI = −0.732 to −0.133, *p* = 0.005), with overlapping 95% CIs (Figure 5).

**FIGURE 6:**
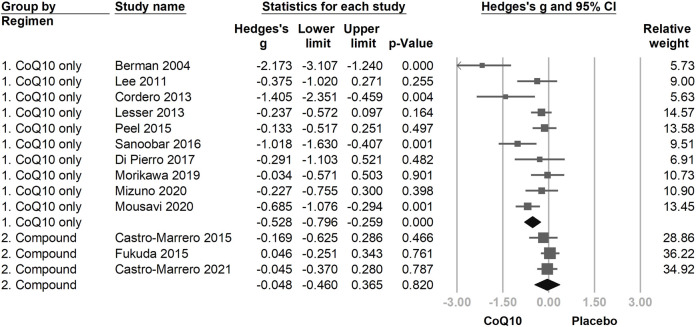
The forest plot of subgroup analysis using the composition of the interventional regimen, including CoQ10 only and CoQ10 compound interventions as the moderators. CoQ10 is effective in reducing fatigue in the CoQ10-only formulation, but not in the mixing compound, which might be associated with the lower dose of CoQ10 used in compound regimens.

Another subgroup analysis was performed according to differences in the evaluated CoQ10 regimens. The group that used only CoQ10 showed a statistically significant fatigue reduction after supplementation (Hedges’ *g* = −0.552, 95% CI = −0.863 to −0.241, and *p* = 0.001). However, the effect of fatigue reduction in the group using CoQ10 compounds (Figure 6) was trivial and statistically insignificant as compared with the placebo group (Hedges’ *g* = −0.028, 95% CI = −0.225 to 0.170, *p* = 0.781).

**FIGURE 7:**
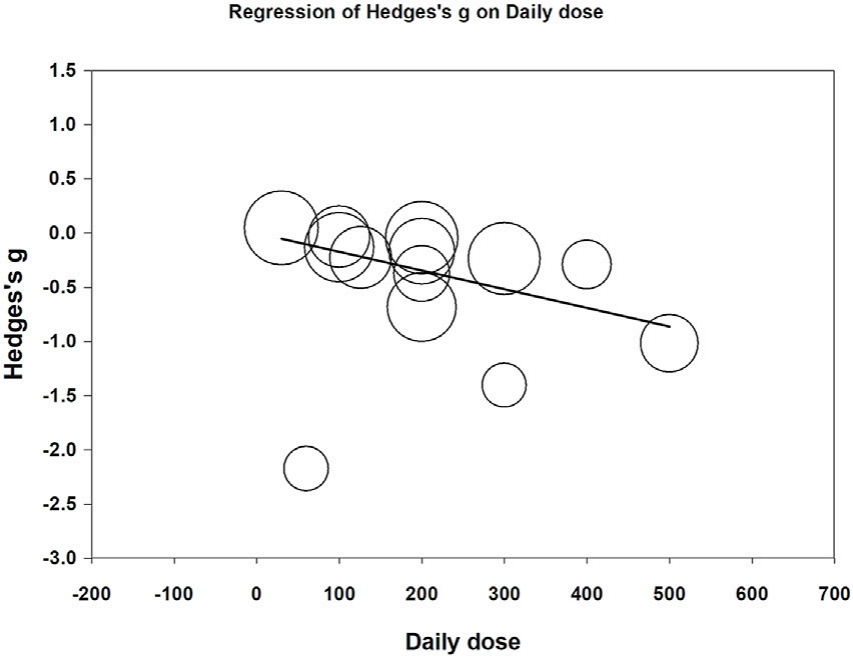
Meta-regression of Hedges’ *g* on daily dose (mg/day). The coefficient was -0.0017 with a *p* value <0.001.

**FIGURE 8:**
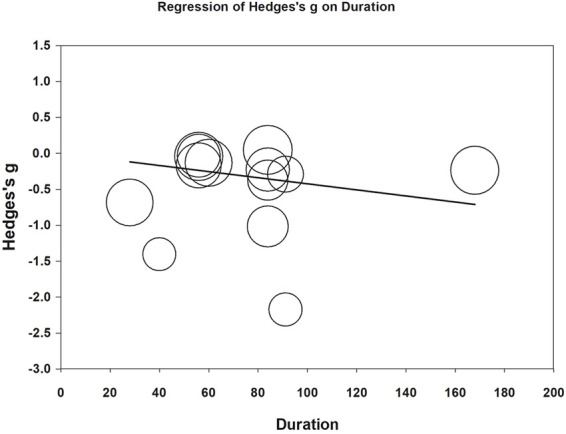
Meta-regression of Hedges’ *g* on treatment duration (day). The coefficient was -0.0042 with a *p* value of 0.007.

Meta-regression was performed to examine whether daily CoQ10 dose and treatment duration could modify effects on fatigue reduction. Both daily doses (coefficient = −0.0017 per mg, *p* < 0.001) and treatment duration (coefficient = −0.0042 per day, *p* = 0.007) correlated with increased fatigue reduction (Figures 7, 8). The funnel plot of the 13 included trials showed some asymmetry in effect size distributions (Supplementary Figure S1). Egger’s regression test showed a *p*-value of 0.008, indicating potential publication bias.

### 3.4 Secondary Outcome: Treatment-Associated Adverse Event Rates

Among the 602 participants treated with CoQ10, only one subject presented with an adverse event (i.e., gastrointestinal upset) and was thus withdrawn from the study conducted by Berman et al. (Berman et al., 2004). The meta-analysis of treatment-associated adverse event rates (Supplementary Figure S2) showed no between-group differences (odds ratio [OR] = 1.05, 95% CI = 0.36 to 3.08, *p* = 0.933, *I* ^*2*^ < 0.01%).

## 4 Discussion

In this meta-analysis, CoQ10 was shown to statistically significantly reduce fatigue, and statistical significance was maintained within sensitivity analyses. The background condition of the participants did not have a statistically significant impact on the direction of the association between the use of CoQ10 and fatigue reduction. We found that CoQ10-only formulations were effective in relieving fatigue, in contrast to CoQ10 compounds. Moreover, an increase in the daily dose and treatment duration of CoQ10 correlated with a better reduction in fatigue. To our knowledge, our study is the first systematic review or meta-analysis to demonstrate that CoQ10 has a mild-to-moderate effect (Hedges, 1981).

Previous meta-analyses have suggested that CoQ10 supplementation can reduce oxidative stress (Sangsefidi et al., 2020) and inflammatory markers (Fan et al., 2017). And in heart failure patients, it is related to lower mortality and higher exercise capacity as compared with placebo-treated group (Lei and Liu, 2017). In 2019, Mehrabani et al. published a systematic review (Mehrabani et al., 2019) reported that CoQ10 was effective against fatigue in patients with myopathy associated with statin use (Fedacko et al., 2013) as well as in patients with fibromyalgia (Cordero et al., 2012a; Cordero et al., 2013; Miyamae et al., 2013; Di Pierro et al., 2017). However, the aforementioned review did not quantify the fatigue-reducing effect of CoQ10. Thus, our meta-analysis was highly novel in addressing a gap in the current literature.

The causes of fatigue are multifactorial (Manjaly et al., 2019), including inflammatory oxidative injury in neurons (Haß et al., 2019; Manjaly et al., 2019) and mitochondrial dysfunction (Filler et al., 2014) and CoQ10 is likewise known to be involved in pathogenic pathways. First, CoQ10 plays a crucial role in inhibiting oxidation of lipid-containing structures, such as cellular membranes and lipoproteins (Aaseth et al., 2021). For example, in a rat model, CoQ10 was found to protect both the peripheral and central nervous systems after passing through the blood-brain barrier (Belousova et al., 2016). CoQ10 may provide widespread protection to neurons through neural pathways connecting the brain to the muscles (Aaseth et al., 2021). Second, CoQ10 regulates the mitochondrial respiratory chain and is an intensively studied enzyme associated with mitochondrial dysfunction (Filler et al., 2014). To this end, Maes et al. compared patients with depression (Maes et al., 2009b) and chronic fatigue syndrome (Maes et al., 2009a) with healthy volunteers and found that CoQ10 deficiency was positively associated with fatigue, whereas serum CoQ10 levels were inversely correlated with fatigue severity (Maes et al., 2009a; Maes et al., 2009b). The above pathways and clinical observations could explain the fatigue-reducing effect of CoQ10 concluded by our meta-analysis.

In the subgroup analysis evaluating healthy participants separately from patients with disease, the directions of the CoQ10-associated effect sizes in both subgroups were consistent. We found that the subgroup of patients demonstrated statistical significance (*p* = 0.004) in terms of the summary effect size, while the healthy subgroup did not show statistical significance (*p* = 0.191); however, this subgroup showed a strong tendency towards positive effects of CoQ10, with demonstrated marginal significance and the upper limit of the 95% CI just crossing zero (Hedges’ *g* = −0.338, 95% CI = −0.844 to 0.168). This could be related to between-group differences in CoQ10 depletion, which might be more severe in patients with diseases than in healthy participants, thus indicating that CoQ10 was more effective in reducing fatigue in the former population within the evaluated studies. Additionally, the number of studies included in the patient subgroup was greater than in the healthy subgroup, which subsequently increased the statistical power and narrowed the pooled 95% CI to facilitate a statistically significant result.

The fatigue-reducing effects of CoQ10 were not statistically significant in the subgroup treated with CoQ10-compounds. We speculated that this might be associated with the lower CoQ10 dose in the evaluated CoQ10 compound regimens (i.e., 30 mg/day (Fukuda et al., 2015) to 200 mg/day (Castro-Marrero et al., 2015; Castro-Marrero et al., 2021)). In contrast, in the subgroup using CoQ10 only (Berman et al., 2004; Lee et al., 2011; Cordero et al., 2013; Lesser et al., 2013; Peel et al., 2015; Sanoobar et al., 2016; Di Pierro et al., 2017; Morikawa et al., 2019; Mizuno et al., 2020; Mousavi et al., 2020), the daily dose ranged from 60 to 500 mg/day with four studies utilizing 300–500 mg/day (Cordero et al., 2013; Lesser et al., 2013; Sanoobar et al., 2016; Di Pierro et al., 2017). Meta-regression also confirmed that the daily dose of CoQ10 was associated with fatigue reduction (Kirkland, 2022; NOW, 2022).

Moreover, we identified a positive relationship between treatment duration and fatigue reduction. In our included studies, the longest period of CoQ10 supplementation was 6 months. As fatigue is a complicated disorder involving both psychological and physiological pathways, a sufficient period of intervention is needed to restore CoQ10 from chronic depletion status. Our speculation was evident in the results of a prior study (Hershey et al., 2007), which indicated that it takes approximately 3 months for CoQ10 supplementation to take effect in patients with chronic illness and CoQ10 deficiency. In the future, a prospective trial may be necessary to examine when ceiling effects are expected within CoQ10 interventions.

We also tried to explore if the fatigue-reducing effect of CoQ10 is related to the formulation, bioavailability or the patient’s disease. However, no specific pattern can be found. For example, in the three studies (Berman et al., 2004; Cordero et al., 2013; Sanoobar et al., 2016) with the Hedges’ *g* < −1.0, the formulations and manufacturers of CoQ10 were all different. Furthermore, Cordero et al. (Cordero et al., 2013) studied patients with fibromyalgia and the Hedges’ *g* was −1.405. Nonetheless, Di Pierro et al. (Di Pierro et al., 2017) studied the same fibromyalgia condition just got an Hedges’ *g* of −0.291. The effects of the formulation, bioavailability and disease might need more studies to conclude.

Coenzyme Q10 is a well-studied substance with a well-documented safety level of 1,200 mg/day per person (Hidaka et al., 2008). Moreover, evidence from pharmacokinetic studies suggests that exogenous CoQ10 does not influence the biosynthesis of endogenous CoQ10 and is less likely to accumulate in plasma or tissues after the cessation of supplementation (Bhagavan and Chopra, 2007; Miles, 2007). The high safety profile in the latter research was also compatible with our findings that, even at relatively high daily doses of 300–500 mg/day, the adverse event rate in the CoQ10 group was never higher than in the placebo group.

This study has several limitations. First, the fatigue scales used in different trials differed, which may have contributed to heterogeneity. Thus, in this study, we standardized measurements using Hedges’ *g*, employed a random-effects model to pool the studies, and conducted a subgroup analysis in accordance with the standard practice for addressing heterogeneity suggested by the Cochrane Handbook (Higgins et al., 2021a; Higgins et al., 2021b; Page et al., 2021b; Deeks et al., 2021; Tsai et al., 2021). Second, the daily dose and intervention duration were different in each trial, which may have contributed to variations in the estimated effects. Therefore, meta-regressions were performed to examine whether a linear relationship exists between the aforementioned factors and fatigue reduction. Third, these studies did not follow the participants after the cessation of CoQ10 supplementation to investigate how long the fatigue-reducing effect lasted, which serves as an intriguing topic to explore in future trials.

In conclusion, CoQ10 demonstrated a statistically significant fatigue-alleviating effect as compared with the evaluated placebos. The effect was statistically significantly correlated with daily dose and treatment duration. Future studies are needed to investigate the lasting effects of CoQ10 on fatigue reduction after the discontinuation of supplementation.

## Data Availability

The original contributions presented in the study are included in the article/Supplementary Material, further inquiries can be directed to the corresponding author.
