# Supplementary material for: Effectiveness of Coenzyme Q10 Supplementation for Reducing Fatigue: A Systematic Review and Meta-Analysis of Randomized Controlled Trials
Source: Front Pharmacol. 2022 Aug 24;13:883251. doi: 10.3389/fphar.2022.883251 (PMC9449413; doi:10.3389/fphar.2022.883251)
Supplement: Supplementary file 1 [file DataSheet1.pdf]

Part 1: Supplementary Figures

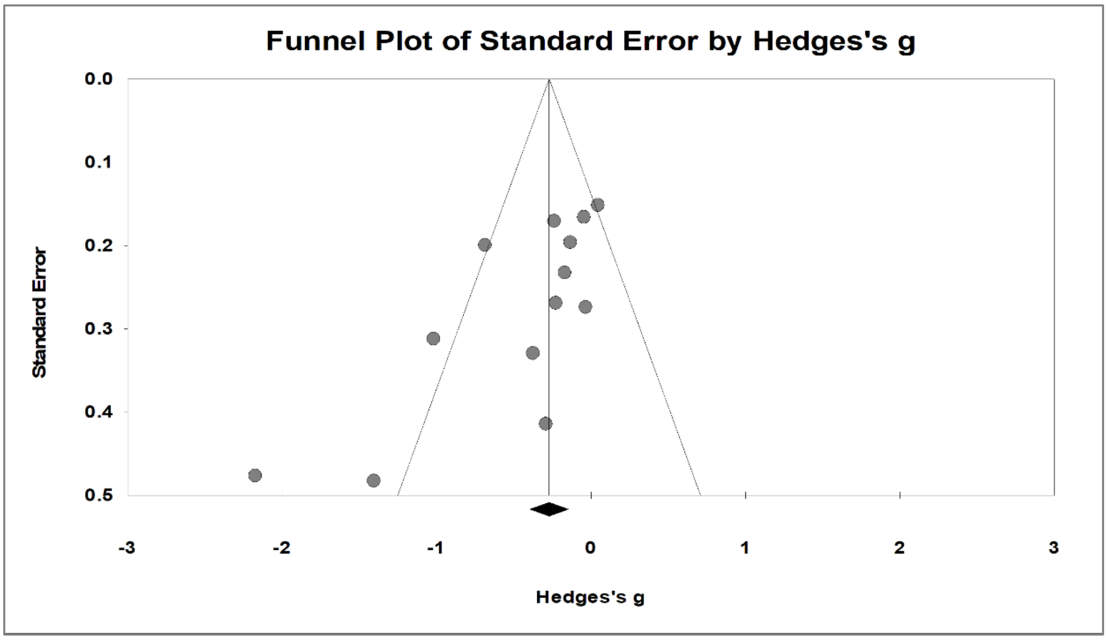

Figure S1. The funnel plot of included trials showed asymmetric distribution. The *p* value of the Egger's test was 0.008, indicating potential publication bias.

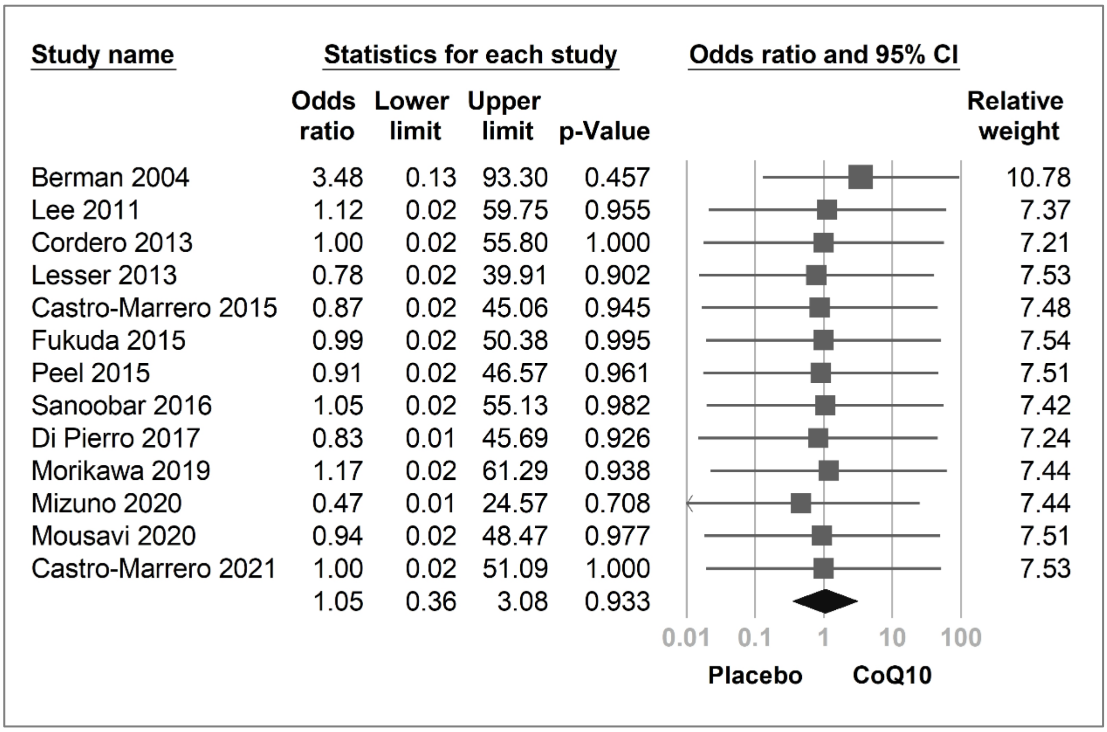

Figure S2. Forest plot of treatment-related adverse event rates. In all 13 trials and 602 participants treated with CoQ10, only one CoQ10-related gastrointestinal upset case was noted in Berman 2004 study. The case also withdrew from the trial.

Part 2: Supplementary Tables

Table S1. PRISMA Checklist

Section and Topic	#	Checklist item	Location
**TITLE**			
Title	1	Identify the report as a systematic review.	Title
**ABSTRACT**			
Abstract	2	See the PRISMA 2020 for Abstracts checklist.	Abstract
**INTRODUCTION**			
Rationale	3	Describe the rationale for the review in the context of existing knowledge.	Introduction
Objectives	4	Provide an explicit statement of the objective(s) or question(s) the review addresses.	Introduction
**METHODS**			
Eligibility criteria	5	Specify the inclusion and exclusion criteria for the review and how studies were grouped for the syntheses.	Methods
Information sources	6	Specify all databases, registers, websites, organisations, reference lists and other sources searched or consulted to identify studies. Specify the date when each source was last searched or consulted.	Methods
Search strategy	7	Present the full search strategies for all databases, registers and websites, including any filters and limits used.	Methods, Table S2
Selection process	8	Specify the methods used to decide whether a study met the inclusion criteria of the review, including how many reviewers screened each record and each report retrieved, whether they worked independently, and if applicable, details of automation tools used in the process.	Methods
Data collection process	9	Specify the methods used to collect data from reports, including how many reviewers collected data from each report, whether they worked independently, any processes for obtaining or confirming data from study investigators, and if applicable, details of automation tools used in the process.	Methods, Table S4
Data items	10a	List and define all outcomes for which data were sought. Specify whether all results that were compatible with each outcome domain in each study were sought (e.g., for all measures, time points, analyses), and if not, the methods used to decide which results to collect.	Methods, Table S4
	10b	List and define all other variables for which data were sought (e.g., participant and intervention characteristics, funding sources). Describe any assumptions made about any missing or unclear information.	Methods<br>Table 1-2, Table S4
Study risk of bias assessment	11	Specify the methods used to assess risk of bias in the included studies, including details of the tool(s) used, how many reviewers assessed each study and whether they worked independently, and if applicable, details of automation tools used in the process.	Methods
Effect measures	12	Specify for each outcome the effect measure(s) (e.g., risk ratio, mean difference) used in the synthesis or presentation of results.	Methods
Synthesis methods	13a	Describe the processes used to decide which studies were eligible for each synthesis (e.g., tabulating the study intervention characteristics and comparing against the planned groups for each synthesis (item #5)).	Methods, Figure 1,<br>Table 1-2, Table S3
	13b	Describe any methods required to prepare the data for presentation or synthesis, such as handling of missing summary statistics, or data conversions.	Methods, Table S4
	13c	Describe any methods used to tabulate or visually display results of individual studies and syntheses.	Methods
	13d	Describe any methods used to synthesize results and provide a rationale for the choice(s). If meta-analysis was performed, describe the model(s), method(s) to identify the presence and extent of statistical heterogeneity, and software package(s) used.	Methods
	13e	Describe any methods used to explore possible causes of heterogeneity among study results (e.g., subgroup analysis, meta-regression).	Methods

	13f	Describe any sensitivity analyses conducted to assess robustness of the synthesized results.	Methods
Reporting bias assessment	14	Describe any methods used to assess risk of bias due to missing results in a synthesis (arising from reporting biases).	Methods, Figure 2, Table 3
Certainty assessment	15	Describe any methods used to assess certainty (or confidence) in the body of evidence for an outcome.	Methods
**RESULTS**			
Study selection	16a	Describe the results of the search and selection process, from the number of records identified in the search to the number of studies included in the review, ideally using a flow diagram.	Results, Figure 1, Table S2-S3
	16b	Cite studies that might appear to meet the inclusion criteria, but which were excluded, and explain why they were excluded.	Results, Table S3
Study characteristics	17	Cite each included study and present its characteristics.	Results, Table 1-2
Risk of bias	18	Present assessments of risk of bias for each included study.	Figure 2, Table 3
Results of individual studies	19	For all outcomes, present, for each study: (a) summary statistics for each group (where appropriate) and (b) an effect estimates and its precision (e.g., confidence/credible interval), ideally using structured tables or plots.	Figure 3-8, Figure S1-S2
Results of syntheses	20a	For each synthesis, briefly summarise the characteristics and risk of bias among contributing studies.	Results, Table 3
	20b	Present results of all statistical syntheses conducted. If meta-analysis was done, present for each the summary estimate and its precision (e.g., confidence/credible interval) and measures of statistical heterogeneity. If comparing groups, describe the direction of the effect.	Results, Figure 3-8, Figure S1-S2
	20c	Present results of all investigations of possible causes of heterogeneity among study results.	Results, Figure 3-8, Figure S1-S2
	20d	Present results of all sensitivity analyses conducted to assess the robustness of the synthesized results.	Results, Figure 4
Reporting biases	21	Present assessments of risk of bias due to missing results (arising from reporting biases) for each synthesis assessed.	Figure 2, Table 3
Certainty of evidence	22	Present assessments of certainty (or confidence) in the body of evidence for each outcome assessed.	Figure 3-8, Figure S1-S2
**DISCUSSION**			
Discussion	23a	Provide a general interpretation of the results in the context of other evidence.	Discussion
	23b	Discuss any limitations of the evidence included in the review.	Discussion
	23c	Discuss any limitations of the review processes used.	Discussion
	23d	Discuss implications of the results for practice, policy, and future research.	Discussion
**OTHER INFORMATION**			
Registration and protocol	24a	Provide registration information for the review, including register name and registration number, or state that the review was not registered.	Methods
	24b	Indicate where the review protocol can be accessed, or state that a protocol was not prepared.	Methods, Table S2-S3
	24c	Describe and explain any amendments to information provided at registration or in the protocol.	Methods, Table S2-S3
Support	25	Describe sources of financial or non-financial support for the review, and the role of the funders or sponsors in the review.	Funding
Competing interests	26	Declare any competing interests of review authors.	Conflicts of Interest
Availability of data, code and other materials	27	Report which of the following are publicly available and where they can be found: template data collection forms; data extracted from included studies; data used for all analyses; analytic code; any other materials used in the review.	Results, Table S2-S4

Table S2. Keywords and search results in different databases

Database	Keyword	Filter	Date	Results
PubMed	('Q10' OR 'Q 10' OR 'CoQ10' OR 'Coenzyme Q10' OR 'ubiquinol-10' OR 'ubiquinol' OR 'ubiquinone') AND ('fatigue' OR 'chronic fatigue syndrome' OR 'tiredness')	NA	January 16, 2022	402
Embase	('Q10' OR 'Q 10' OR 'CoQ10' OR 'Coenzyme Q10' OR 'ubiquinol-10' OR 'ubiquinol' OR 'ubiquinone') AND ('fatigue' OR 'chronic fatigue syndrome' OR 'tiredness')	Title Abstract Keyword	January 16, 2022	213
Cochrane CENTRAL	('Q10' OR 'Q 10' OR 'CoQ10' OR 'Coenzyme Q10' OR 'ubiquinol-10' OR 'ubiquinol' OR 'ubiquinone') AND ('fatigue' OR 'chronic fatigue syndrome' OR 'tiredness')	Title Abstract Keyword	January 16, 2022	277
Web of Science	('Q10' OR 'Q 10' OR 'CoQ10' OR 'Coenzyme Q10' OR 'ubiquinol-10' OR 'ubiquinol' OR 'ubiquinone') AND ('fatigue' OR 'chronic fatigue syndrome' OR 'tiredness')	NA	January 16, 2022	1106
ClinicalTrials.gov	('Q10' OR 'Q 10' OR 'CoQ10' OR 'Coenzyme Q10' OR 'ubiquinol-10' OR 'ubiquinol' OR 'ubiquinone') AND ('fatigue' OR 'chronic fatigue syndrome' OR 'tiredness')	Condition or disease	January 16, 2022	10

NA: not applied

Table S3. Excluded studies and reasons

Citations	Reasons
Cordero, M.D.; Cano-García, F.J.; Alcocer-Gómez, E.; De Miguel, M.; Sánchez-Alcázar, J.A. Oxidative Stress Correlates with Headache Symptoms in Fibromyalgia: Coenzyme Q10 Effect on Clinical Improvement. *PLOS ONE* **2012**, 7, e35677, doi:10.1371/journal.pone.0035677.	Not a randomized trial
Langsjoen, P.H.; Langsjoen, J.O.; Langsjoen, A.M.; Lucas, L.A. Treatment of statin adverse effects with supplemental Coenzyme Q10 and statin drug discontinuation. *Biofactors* **2005**, 25, 147-152, doi:10.1002/biof.5520250116.	Not a randomized trial
Langsjoen, P.H.; Langsjoen, J.O.; Langsjoen, A.M.; Rosenfeldt, F. Statin-Associated Cardiomyopathy Responds to Statin Withdrawal and Administration of Coenzyme Q(10). *Perm J* **2019**, 23, doi:10.7812/tpp/18.257.	Not a randomized trial
Menon, R.; Cribb, L.; Murphy, J.; Ashton, M.M.; Oliver, G.; Dowling, N.; Turner, A.; Dean, O.; Berk, M.; Ng, C.H.; et al. Mitochondrial modifying nutrients in treating chronic fatigue syndrome: A 16-week open-label pilot study. *Advances in Integrative Medicine* **2017**, 4, 109-114, doi:10.1016/j.aimed.2017.11.001.	Not a randomized trial
Moccia, M.; Capacchione, A.; Lanzillo, R.; Carbone, F.; Micillo, T.; Perna, F.; De Rosa, A.; Carotenuto, A.; Albero, R.; Matarese, G.; et al. Coenzyme Q10 supplementation reduces peripheral oxidative stress and inflammation in interferon-β1a-treated multiple sclerosis. *Ther Adv Neurol Disord* **2019**, 12, 1756286418819074, doi:10.1177/1756286418819074.	Not a randomized trial
Cordero, M.D.; Santos-García, R.; Bermejo-Jover, D.; Sánchez-Domínguez, B.; Jaramillo-Santos, M.R.; Bullón, P. Coenzyme Q10 in salivary cells correlate with blood cells in Fibromyalgia: improvement in clinical and biochemical parameter after oral treatment. *Clin Biochem* **2012**, 45, 509-511, doi:10.1016/j.clinbiochem.2012.02.001.	Not a randomized trial
Miyamae, T.; Seki, M.; Naga, T.; Uchino, S.; Asazuma, H.; Yoshida, T.; Iizuka, Y.; Kikuchi, M.; Imagawa, T.; Natsumeda, Y.; et al. Increased oxidative stress and coenzyme Q10 deficiency in juvenile fibromyalgia: amelioration of hypercholesterolemia and fatigue by ubiquinol-10 supplementation. *Redox Rep* **2013**, 18, 12-19, doi:10.1179/1351000212y.0000000036.	Not a randomized trial
Gharahdaghi, N.; Shabkhiz, F.; Azarboo, E.; Keyhanian, A. The Effects of Daily Coenzyme Q 10 Supplementation on VO 2max , vVO 2max and Intermittent Exercise Performance in Soccer Players. *Life Science Journal* **2013**, 10, 22-28.	Focused on muscle fatigue, rather than generalized fatigue.
Gökbel, H.; Gül, I.; Belviranl, M.; Okudan, N. The effects of coenzyme Q10 supplementation on performance during repeated bouts of supramaximal exercise in sedentary men. *J Strength Cond Res* **2010**, 24, 97-102, doi:10.1519/JSC.0b013e3181a61a50.	Focused on muscle fatigue, rather than generalized fatigue.
Negro, M.; Perna, S.; Spadaccini, D.; Castelli, L.; Calanni, L.; Barbero, M.; Cescon, C.; Rondanelli, M.; D'Antona, G. Effects of 12 Weeks of Essential Amino Acids (EAA)-Based Multi-Ingredient Nutritional Supplementation on Muscle Mass, Muscle Strength, Muscle Power and Fatigue in Healthy Elderly Subjects: A Randomized Controlled Double-Blind Study. *J Nutr Health Aging* **2019**, 23, 414-424, doi:10.1007/s12603-019-1163-4.	Focused on muscle fatigue, rather than generalized fatigue.
Suzuki, Y.; Nagato, S.; Sakuraba, K.; Morio, K.; Sawaki, K. Short-term ubiquinol-10 supplementation alleviates tissue damage in muscle and fatigue caused by strenuous exercise in male distance runners. *Int J Vitam Nutr Res* **2021**, 91, 261-270, doi:10.1024/0300-9831/a000627.	Focused on muscle fatigue, rather than generalized fatigue.

Mizuno, K.; Tanaka, M.; Nozaki, S.; Mizuma, H.; Ataka, S.; Tahara, T.; Sugino, T.; Shirai, T.; Kajimoto, Y.; Kuratsune, H.; et al. Antifatigue effects of coenzyme Q10 during physical fatigue. *Nutrition* **2008**, 24, 293-299, doi:10.1016/j.nut.2007.12.007.	Focused on muscle fatigue, rather than generalized fatigue.
Fedacko, J.; Pella, D.; Fedackova, P.; Hänninen, O.; Tuomainen, P.; Jarcuska, P.; Lopuchovsky, T.; Jedlickova, L.; Merkovska, L.; Littarru, G.P. Coenzyme Q(10) and selenium in statin-associated myopathy treatment. *Can J Physiol Pharmacol* **2013**, 91, 165-170, doi:10.1139/cjpp-2012-0118.	Focused on muscle fatigue, rather than generalized fatigue.
Iwase, S.; Kawaguchi, T.; Yotsumoto, D.; Doi, T.; Miyara, K.; Odagiri, H.; Kitamura, K.; Ariyoshi, K.; Miyaji, T.; Ishiki, H.; et al. Efficacy and safety of an amino acid jelly containing coenzyme Q10 and L-carnitine in controlling fatigue in breast cancer patients receiving chemotherapy: a multi-institutional, randomized, exploratory trial (JORTC-CAM01). *Support Care Cancer* **2016**, 24, 637-646, doi:10.1007/s00520-015-2824-4.	Used 'no intervention' as control group, rather than matching placebo.
Schweiger, V.; Secchettin, E.; Castellani, C.; Martini, A.; Mazzocchi, E.; Picelli, A.; Polati, E.; Donadello, K.; Valenti, M.T.; Dalle Carbonare, L. Comparison between Acupuncture and Nutraceutical Treatment with Migratens(®) in Patients with Fibromyalgia Syndrome: A Prospective Randomized Clinical Trial. *Nutrients* **2020**, 12, doi:10.3390/nu12030821.	Used acupuncture as control group, rather than matching placebo.
Singh, R.B.; Neki, N.S.; Kartikey, K.; Pella, D.; Kumar, A.; Niaz, M.A.; Thakur, A.S. Effect of coenzyme Q10 on risk of atherosclerosis in patients with recent myocardial infarction. *Mol Cell Biochem* **2003**, 246, 75-82.	Utilized a binary outcome (yes or no) rather than a continuous scale for fatigue quantification.
Kumar, A.; Singh, R.B.; Saxena, M.; Niaz, M.A.; Josh, S.R.; Chattopadhyay, P.; Mechirova, V.; Pella, D.; Fedacko, J. Effect of carni Q-gel (ubiquinol and carnitine) on cytokines in patients with heart failure in the Tishcon study. *Acta Cardiol* **2007**, 62, 349-354, doi:10.2143/ac.62.4.2022278.	Utilized a binary outcome (yes or no) rather than a continuous scale for fatigue quantification.
Fukuda, S.; Nojima, J.; Kajimoto, O.; Yamaguti, K.; Nakatomi, Y.; Kuratsune, H.; Watanabe, Y. Ubiquinol-10 supplementation improves autonomic nervous function and cognitive function in chronic fatigue syndrome. *Biofactors* **2016**, 42, 431-440, doi:10.1002/biof.1293.	No available data of pre- and post-intervention fatigue assessment nor changes in fatigue scores.
Gomez-Centeno, A.; Ramentol, M.; Gonzalez, M.J.; Alegre, C. AB0952 COENZYME Q10, TRYPTOPHAN AND MAGNESIUM: A NUTRITIONAL SUPPLEMENT IN THE TREATMENT OF FIBROMYALGIA SYMPTOMS. *Annals of the Rheumatic Diseases* **2020**, 79, 1773-1774, doi:10.1136/annrheumdis-2020-eular.5531.	No available data of pre- and post-intervention fatigue assessment nor changes in fatigue scores.
Castro-Marrero, J.; Sáez-Francàs, N.; Segundo, M.J.; Calvo, N.; Faro, M.; Aliste, L.; Fernández de Sevilla, T.; Alegre, J. Effect of coenzyme Q10 plus nicotinamide adenine dinucleotide supplementation on maximum heart rate after exercise testing in chronic fatigue syndrome - A randomized, controlled, double-blind trial. *Clin Nutr* **2016**, 35, 826-834, doi:10.1016/j.clnu.2015.07.010.	Overlapping participants with the author's another publication (18), which is already enrolled in our meta-analysis.

Table S4. Details of data extraction from included randomized controlled trials

First Author & Year	Details of data extraction from included trials
Berman 2004	1. The age and sex of allocated participants were from Materials and Methods - Patients - Second paragraph.<br>2. The treatment-effect data was extracted from 'Results-Anamnesis' (meaning a record of medical history) paragraph, not from Figure 2 measurement.<br>3. Per-protocol participant numbers of intervention and control groups were not provided in the article. Only the total number (27) was presented. We contacted the corresponding author by email but received no response. The participant numbers of both groups were estimated according to Results' first paragraph as 13 for CoQ10 and 14 for placebo.<br>4. The paired *p* value of placebo was estimated by median difference. The placebo group showed larger median difference than CoQ10 group, suggesting the *p* value should be similar ($p < 0.001$).<br>5. The data was presented as median, with no interquartile range provided. According to the Cochrane Handbook, we used the median as mean.<br>6. The range of age was provided instead of standard deviation. The data was converted according to Hozo et al.'s suggestion. For moderately sized samples ($15 < n \leq 70$), range/4 is the best estimator for the standard deviation. For large samples ($n > 70$), the formula range/6 gives the best estimator for the standard deviation. (Hozo et al., 2005)
Lee 2011	1. Participants' age and sex were from Table 1.<br>2. The treatment-effect data was extracted from Figure 2 measurement. Notice the y-axis is from 20.0, rather than 0.0. Per-protocol participant numbers were from Table 2.
Cordero 2013	1. All-female study. Participants' age and number were from Supplementary Table S1.<br>2. The data was extracted from Figure 2B measurement. To improve measurement precision, Figure 2B was magnified and printed out and then measured.
Lesser 2013	1. All-female study. Participants' age and number were from Table 1.<br>2. The range of age was provided instead of standard deviation. The data was converted according to Hozo et al.'s suggestion. For moderately sized samples ($15 < n \leq 70$), range/4 is the best estimator for the standard deviation. For large samples ($n > 70$), the formula range/6 gives the best estimator for the standard deviation. (Hozo et al., 2005)<br>3. The treatment-effect data was extracted from Table 2 POMS-F. Week 0 total participants data was used as the pre-intervention baseline. Per-protocol participant numbers of each group were from week 24 data.
Castro-Marrero 2015	1. All-female study. Participants' age and number were from Figure 1 and Results - Study participants.<br>2. The treatment-effect data was from Table 2 FIS total score.
Fukuda 2015	1. The age, sex, allocated and per-protocol participant numbers of both groups were from Figure 1 and Table 1. Note the N of placebo drink was inconsistent between Figure 1 (86) and Table 1 (87). After cross-validation, Figure 1 (86) was considered as the correct one.<br>2. The treatment-effect data was extracted from Table 2 Fatigue score / fatigue / week 0 & week 12. Fatigue (VAS) was for acute condition. Chronic fatigue (Fatigue score) was the primary outcome, which was mentioned in Methods - Study outcomes - paragraph 4. Note the effect direction was positive, which means the CoQ10 drink reduced less fatigue than placebo (Placebo: -0.90, CoQ10 drink: -0.69).
Peel 2015	1. The allocated and analyzed per-protocol participant numbers were from Figure 1. The N of CoQ10 group in Table 1 (52) was not consistent with Table 2 and Figure 1 (54). After cross-validation, we used Table 2 and Figure 1 for data extraction.<br>2. The age and sex were from Table 1.<br>3. The treatment-effect data was extracted from Table 2 MAF.<br>4. The data was presented in standard errors, which were then converted into standard deviations according to Cochrane Handbook suggestions.<br>5. In this study, two fatigue scales were used for assessment. We decided to use MAF rather than FSS, because MAF is more comprehensive than FSS and MAF is the primary outcome of the study as described in 2. Method - 2.4 Assessment of fatigue.

Sanoobar 2016	1. The age, sex, allocated and analyzed per-protocol participant numbers of both groups were from Table 1 and Figure 1.<br>2. The treatment-effect data was from Table 3 FSS.
Di Pierro 2017	1. All-female study. Participants' age and numbers were from Table 1.<br>2. The treatment-effect data was extracted from Table 1 and Table 2 FACIT row.<br>3. Though the authors stated in the note of Table 1 that all values were expressed as the median ± standard deviation, we consider it as a typographical error and it should be mean ± standard deviation. Two reasons supported the decision. First, the decimals demonstrated numbers like 0.9 and 0.1, which are typical for mean values not median ones. Second, the median is usually accompanied by interquartile range, rather than standard deviation. The standard deviation was usually used with the mean.<br>4. The direction of the FACIT score was determined according to Results - second paragraph - 7th point description, which stated that 22.4% reduction of FACIT score means the improvement of the fatigue symptom. In the article, reference 25 was used to support their use of FACIT score. (Chandran et al., 2007)<br>5. This is a cross-over study. The first 3-months treatment before cross-over was used for data extraction.
Morikawa 2019	1. This article was published in a Japanese journal which is not indexed by PubMed and not accessible by available library resource. The study was found in Embase. The full text PDF can be bought online.<br>2. The age, sex and allocated participant numbers of both groups were from Table 1.<br>3. The treatment-effect data was from Table 2 - right column - fourth data pair - 8-week change from baseline values. The analyzed per-protocol participant numbers of both groups were also from Table 2.<br>4. The data was presented in standard errors, which were then converted into standard deviations according to Cochrane Handbook suggestions.
Mizuno 2020	1. The trial had three arms, including placebo, 100 mg/day CoQ10 and 150 mg/day CoQ10. The two treatment arms were combined to create a single pair-wise comparison according to the Cochrane Handbook.<br>2. The age, sex and participant number of both groups were from Table 1.<br>3. The data of pre- and post-intervention difference was from Table 2 - Post-Task fatigue score (week 12).<br>4. Post-task fatigue score was used since it can better represent the real-life situation than pre-task one.
Mousavi 2020	1. This trial was published in a British journal not indexed by PubMed. The article was found in Embase.<br>2. The age, sex and analyzed per-protocol participant number of both groups were from Table 2. The allocated participant number was from Figure 1.<br>3. The treatment-effect data was from Table 3 - Fatigue.
Castro-Marrero 2021	1. All-female study. The age and analyzed per-protocol participant number of both groups were from Table 1. The allocated participant number was from Figure 1.<br>2. The treatment-effect data was from Table 2 - Total FIS-40 score - Baseline - 8 weeks. The effect size direction is negative, meaning CoQ10+NADH reduced more fatigue than placebo (CoQ10+NADH: -2.54, Placebo: -1.45).

BFI: Brief Fatigue Inventory; Cochrane Handbook: Deeks et al., 2021; Higgins et al., 2021a; Higgins et al., 2021b; CoQ10: coenzyme Q10; FACIT: Functional Assessment of Chronic Illness Therapy; FIS: Fatigue Impact Scale; FSS: Fatigue Severity Scale; MAF: Multidimensional Assessment of Fatigue; N: participant number; POMS-F: Profile of Moods States Fatigue subscale; Ref: reference number.
